# The role of emotional functioning in the relationship between health anxiety and cyberchondria

**DOI:** 10.1007/s12144-022-04126-3

**Published:** 2022-12-19

**Authors:** Agata Błachnio, Aneta Przepiórka, Paweł Kot, Andrzej Cudo, Stanisława Steuden

**Affiliations:** Institute of Psychology, The John Paul II Catholic University of Lublin, Al. Racławickie 14, 20-950 Lublin, Poland

**Keywords:** Cyberchondria, Health anxiety, Optimism vs. pessimism, Distress, Difficulties in emotion regulation

## Abstract

Access to health information can have positive effects in the form of increased health awareness. However, self-diagnosis based on information available on websites can be dangerous, causing distress as an increasing amount of time is spent on searching. The main aim of the study was to determine the role of emotional functioning in the relationship between health anxiety and cyberchondria. The study included *N* = 615 participants aged 18 to 83 years (*M* = 43.86, *SD* = 14.57). The authors used the Cyberchondria Severity Scale, the Short Health Anxiety Inventory, the Life Orientation Test, the Difficulties in Emotion Regulation Scale, and the Kessler 6 Psychological Distress Scale. The results demonstrate that health anxiety is significantly related to cyberchondria and that pessimism and difficulties in emotion regulation act as mediators in this relationship.

**Supplementary Information:**

The online version contains supplementary material available at 10.1007/s12144-022-04126-3.

## Introduction

The Internet is currently becoming an increasingly popular source of health information. With virtually unrestricted access to the Internet, people more and more often obtain information about their health on-line (Huberty et al., 2013; Singh & Brown, 2016). They search for it not only to complement specialists’ diagnosis; self-diagnosis based on information obtained from the Internet is becoming an increasingly frequent phenomenon. Access to health information can have positive effects by increasing health awareness (McElroy et al., 2019). However, self-diagnosis based on information available on-line can be dangerous, which is why this phenomenon is sometimes discussed as disturbing (Lopez-Fernandez, 2019; McElroy & Shevlin, 2014). Searching for health information may lead to distress and health anxiety caused by searching itself and by devoting an increasing amount of time to this activity (McElroy et al., 2019). In the literature, cyberchondria is defined as severe concern about one’s health associated with problematic Internet use and obsessive–compulsive symptoms (e.g., Doherty-Torstrick et al., 2016; Starcevic et al., 2019). It is also sometimes referred to as the new psychopathology of the twenty-first century (Starcevic & Aboujaoude, 2015). Various international studies indicate that between 40 and 80% of Internet users have searched for information about their physical or mental health on the Internet (Maftei & Holman, 2020; McElroy et al., 2019; Vismara et al., 2020). Naturally, not every search for information about the symptoms one is experiencing indicates cyberchondria. It is natural for people to worry about their health. The worry becomes problematic, however, when the severity of the fear of illness and the frequency of information seeking become excessive and begin to interfere with normal functioning (Bati et al., 2018). Cyberchondria is defined as excessive search of the Internet for information about medical symptoms and ailments which produces or exacerbates the experience of severe anxiety about one's health (Doherty-Torstrick et al., 2016) and can even take a dispositional form (Starcevic et al., 2019).

McElroy et al. (2019) distinguish two aspects of cyberchondria: behavioral and emotional. The behavioral aspect refers to how a person searches for information about their disease and how they try to assuage their anxiety, while the emotional aspect corresponds to anxiety or fear caused by searching itself and by the inability to control one’s behavior when searching for information about a particular disease. According to McElroy et al. (2019), searching for medical information because of fear for one's health has four dimensions: excessiveness (repeated searching), compulsion (searching that interferes with other aspects of on-line and off-line life), distress (negative emotional reactions experienced when searching for information about diseases on the Internet), and reassurance (seeking professional medical advice). This is reflected in their short-form version of the Cyberchondria Severity Scale (CSS-12), used to diagnose cyberchondria. The above four dimensions are related to obsessive–compulsive disorders, the category cyberchondria belongs to (Norr et al., 2015; Vismara et al., 2020).

### Health anxiety and cyberchondria

Health is, arguably, the most important resource of the human being (Kabene et al., 2006). That being so, many individuals who are not currently ill experience a natural concern for their health, both physical and mental (Morales et al., 2013). However, when concern for one's health becomes pathological and when the belief that one has symptoms of a severe disease persists despite specialists' assurances that there is no real reason to worry, it is legitimate to speak of a high level of health anxiety (Lucock & Morley, 1996). Health anxiety is an obsessive and irrational worry that stems from a fear of developing a serious physical or mental illness (Salkovskis et al., 2003). Sometimes it consists in misinterpreting minor or normal mental or somatic signals from the body as serious symptoms of a disease (Asmundson et al., 2010). Research results indicate that health anxiety is a dimensional construct (McMullan et al, 2019). One end of the continuum is a complete lack of care about one's health. The other end is excessive health anxiety in the form of hypochondriasis, a persistent unfounded belief that one has at least one serious progressive disease.

Research revealed that individuals experiencing a higher level of anxiety considered themselves to be more exposed to disease and devoted more attention to information concerning health in general (Hadjistavropoulos et al., 1998). Studies have also shown a relationship between cyberchondria and health anxiety (Bati et al., 2018; Baumgartner & Hartmann, 2011; McElroy et al., 2019; Starcevic & Berle, 2013). Health anxiety motivates many people to look for information about their health and potential diseases in on-line sources. However, as studies show (Doherty-Torstrick et al., 2016; Fergus & Russell, 2016; Barke et al., 2016), excessive use of the Internet for self-diagnosis does not only fails to reduce anxiety but may actually intensify it.

Negative emotional states, such as worrying about one's health or feeling threatened, have a particular cognitive and emotional structure. According to Salkovskis et al. (2003), negative cognitive schemas and dysfunctional beliefs about health and diseases are responsible for the development and maintenance of strong health anxiety. They are related to the processes of rumination and have an automatic (habitual) character, being difficult to realize and control (Verplanken et al., 2007); arguably, this is why they contribute to the intensive search for information about health-related threats on Internet forums. False cognitive patterns result in selectively searching for information about one's health and in interpreting such information in a way that fuels anxiety (Lucock & Morley, 1996). A person’s level of arousal upon receiving new information about the disease (including information found on the Internet) determines the general readiness to receive it, while the type of emotions currently experienced may have a selective and distorting effect (Morse & Johnson, 1991) and thus strengthen optimism or a sense of threat.

### Pessimism and cyberchondria

Pessimism is the belief that events will develop in an unfavorable way and that one’s goals or wishes are unlikely to be fulfilled. It is a tendency to see only the negative sides of life, to evaluate reality negatively, and to expect failures in the future (Carver et al., 1994). The opposite of pessimism is optimism. Carver and Scheier (2009) postulate the existence of dispositional optimism, defined as a general and relatively stable tendency to expect successful results in the domains of life that one considers important. Optimism performs self-regulatory functions because it has influence on the choice of goals and determines the effort invested in achieving them. As a rule, pessimism and optimism are regarded as opposite poles of the same dimension.

Optimism and pessimism are related to health behavior. Optimism is considered as one of the personal resources associated with caring for both mental and physical health (Carver & Scheier, 2009; Maftei & Holman, 2020). It is related to health-conscious behaviors such as preventive seeking for information about health, preventive measures, and healthy lifestyle using new technologies (Trinkhaus, 2019), but it is negatively associated with cyberchondria (Maftei & Holman, 2020). In the event of an illness, optimism contributes to faster recovery and has a positive effect on compliance with medical advice. However, excessive optimism may lead to ignoring noticeable symptoms and to underestimating the risk of developing a disease (Sharot et al., 2007). By contrast, a pessimistic approach may lead to excessively critical self-diagnosis and to detecting nonexistent disorders. This may increase anxiety and stress in individuals seeking medical information (Trinkhaus, 2019). The research conducted by Bajcar and Babiak (2020) and by Maftei and Holman (2020) identified pessimism as a correlate of cyberchondria.

### Psychological distress and cyberchondria

Psychological distress is a state of intense psychological pain associated with disagreeable feelings or emotions, such as shame, regret, humiliation, despair, loneliness, sense of harm, or fear (Shneidman, 1999). Distress may occur as a reaction to mental disorders (e.g., depression, anxiety, PTSD), traumatic events (such as a child's death), a loss (of an important relationship, a person, a position, money, or health), social exclusion, realizing one's own limitations, a disease, disability, and the awareness of transience and death (Arvidsdotter et al., 2015; Mee et al., 2011). Psychological distress can be considered as a continuum between mental health and mental illness. Distress can significantly contribute to the deterioration of a person’s physical, mental, interpersonal, social, family, and economic functioning (Shneidman, 1999). Studies (Bajcar & Babiak, 2020; Bottesi et al., 2021; Dameery et al., 2021) have indicated a positive relationship between experiencing psychological distress and cyberchondria—individuals high in psychological distress looked for information about their health on the Internet more often than other subjects. In the same studies, psychological distress was found to co-occur with health anxiety.

### Emotional regulation and cyberchondria

Emotional regulation refers to attempts made to change the emotions experienced by stimulating or maintaining emotional experiences or by modifying their frequency, intensity, or duration (Garnefski & Kraaij, 2007). It comprises the following skills: the awareness and understanding of emotions, the acceptance of emotions, the ability to control impulses, the pursuit of goals also despite negative emotions, and the ability to use appropriate emotional regulation strategies in order to flexibly modulate emotional responses in the process of achieving one's goals (Gratz & Roemer, 2004). Jungmann and Witthöft (2020) found that emotional regulation was a protective factor against cyberchondria and health anxiety, although its effect was indirect. In contrast, difficulties with proper emotional regulation are considered a risk factor for problems with functioning in many spheres of life, including the development of addiction to new technologies (Akbari, 2017). Emotional regulation and impulse control deficits create favorable conditions for problematic Internet use (Caplan, 2010), which in turn is strongly associated with cyberchondria (Bottesi et al., 2021). Research demonstrates that dysfunctional emotional regulation strategies such as rumination and catastrophizing are positively correlated with cyberchondria and health anxiety (Fergus & Russell, 2016; Görgen et al., 2014; Jungmann & Witthöft, 2020). Frequently checking medical information concerning one’s ailments on the Internet is meant to bring temporary relief in the event of recurring thoughts and negative emotions (Fergus & Russell, 2016). As in the case of obsessive–compulsive disorders, emotional balance is briefly restored until another increase in focus on health condition accompanied by negative emotional states (Norr et al., 2015).

## The present study

**Fig. 1:**
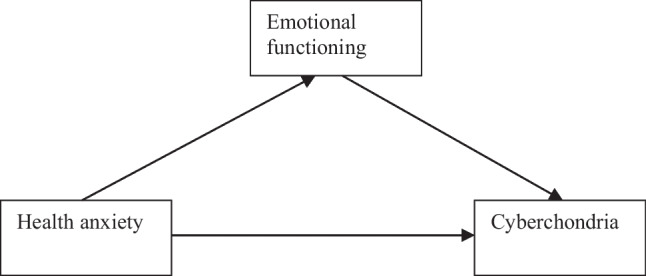
Mediation model of the relationship between health anxiety and cyberchondria

The main aim of the study was to determine the role of emotional functioning in the relationship between health anxiety and cyberchondria (see Fig. 1). We considered health anxiety as composed of two dimensions: (1) health anxiety: illness likelihood and (2) health anxiety: negative consequences of illness (Salkovskis et al., 2002), while cyberchondria was investigated as having four dimensions: compulsion, distress, excessiveness, and reassurance (McElroy et al., 2019). As far as aspects of emotional functioning are concerned, we considered pessimism (Scheier et al., 1994), distress (Kessler et al., 2003), and difficulties in emotion regulation (Gratz & Roemer, 2004).

Based on the literature and previous results, we formulated several hypotheses. A body of research indicated that there was a relationship between cyberchondria and health anxiety (Bati et al., 2018; McElroy et al., 2019; McMullan et al, 2019; Singh & Brown, 2016; Starcevic & Berle, 2013). Therefore, we hypothesized that there would be a positive relationship between health anxiety and cyberchondria (H1). Emotions play a crucial role in experience associated with care for one's health, which means negative emotions can increase health anxiety (Bati et al., 2018). We therefore hypothesized that pessimism (H2), distress (H3), and difficulties in emotion regulation (H4) would act as mediators between health anxiety and cyberchondria.

### Participants

The study included *N* = 615 individuals aged 18 to 83 years (*M* = 43.86, *SD* = 14.57); *n* = 329 of the participants were women. We distinguished five groups according to place of residence: subjects from rural areas (22.6%), from towns with a population up to 20,000 (13.4%), from towns with a population up to 99,000 (22.4%), from cities with up to 500,000 inhabitants (24.2%), and from cities with more than 500,000 inhabitants (17.4%).

For data collection purposes, we used the Ariadna Research Panel (http://www.panelariadna.com). We applied the method known as CAWI (Computer-Assisted Web Interview, being an online interview). Data were collected in February 2020. Participation in the study was voluntary. Participants received shopping vouchers as a form of remuneration. The research was approved by the Research Ethics Board of the Institute of Psychology at the John Paul II Catholic University of Lublin. This article presents partial results from a larger project on cyberchondria. Due to the breadth of the issues addressed in the project and due to the need to ensure consistency, in the present paper we examine only the variables involved in the relationship between health anxiety and cyberchondria via emotional functioning. The remaining results will be reported elsewhere.

### Measures

To measure cyberchondria, we used the short version of the *Cyberchondria Severity Scale* (McElroy et al., 2019; an example item is: “I start to panic when I read online that a symptom I have is found in a rare/serious condition”). The scale consists of 12 items rated on a 5-point Likert scale (1 = *never* to 5 = *almost*). In the Polish version of this measure there are four subscales: Compulsion, Distress, Excessiveness, and Reassurance. Their reliability, measured by Cronbach's α coefficient, is 0.83, 0.87, 0.87, and 0.73, respectively. Compulsion refers to excessive searching for health information on the Internet that interferes with other activities. Distress is the negative emotions experienced when searching for information about diseases on the Internet. Excessiveness means repeatedly looking for information about one's ailments in a variety of on-line sources. Finally, the Reassurance subscale measures the anxiety-driven need to consult a medical expert about information from the Internet.

The *Short Health Anxiety Inventory* (SHAI), adapted into Polish by Kocjan (2016), was used to measure health anxiety (Salkovskis et al., 2002). The questionnaire has 18 items, each consisting of four statements, of which participants choose the one that is the truest about them, e.g., (a) “I do not worry about my health”; (b) “I occasionally worry about my health”; (c) “I spend much of my time worrying about my health”; (d) “I spend most of my time worrying about my health.” The inventory measures two components of health anxiety: illness likelihood and negative consequences of illness. In our study, the reliability of the subscales (Cronbach's α) was 0.72 and 0.93, respectively).

To measure pessimism, we used the 12-item *Life Orientation Test* (LOT-R; Scheier et al., 1994), adapted into Polish by Poprawa and Juczyński and Poprawa (2001). An example item is: “If something can go wrong for me, it will.” Each of the items is rated on a 5-point Likert scale (1 = *completely disagree* to 5 = *completely agree*). The reliability of this method was acceptable, with Cronbach's α = 0.76.

To measure emotion regulation, we administered the *Difficulties in Emotion Regulation Scale* (DERS; Gratz & Roemer, 2004), which consists of 36 items (e.g., “I have no idea how I am feeling”) rated on 5-point Likert scale, from 1 = *almost never* to 5 = *almost always*. Cronbach’s α was 0.87.

Psychological distress was assessed using the *Kessler 6 Psychological Distress Scale* (K6; Kessler et al., 2003), a measure of psychological distress and the outcomes of treatment for common mental health disorders. The K6 consists of six questions about depressive and anxiety symptoms experienced in the most recent four-week period. The questions are answered on a 5-point Likert scale, from *none of the time* (scored as 1) to *all of the time* (scored as 5). The self-report style of the questions assists in the identification of current mental health problems and in deciding whether there is a need for treatment. Cronbach’s α for the K6 in this study was 0.82 for total sample.

### Statistical analysis

Descriptive statistics are presented in the form of arithmetic means and standard deviations for the total sample. We applied the Spearman correlation coefficient to determine the relationships between variables.

To examine the relationships between health anxiety (illness likelihood, negative consequences of illness), distress, pessimism, cyberchondria (excessiveness, distress, reassurance, compulsion), and difficulties in emotion regulation, we performed a path analysis using the maximum likelihood method with Satorra–Bentler adjustment (Satorra & Bentler, 1994). The Satorra-Bentler adjustment was applied because there was a violation of multivariate normal distribution (Mardia’s multivariate skewness test [χ^2^_(*df* = 165)_ = 757.64, *p* < 0.001]; Mardia’s multivariate kurtosis test [χ^2^_(*df* = 1)_ = 358.72, *p* < 0.001); Henze–Zirkler’s consistent test [χ^2^_(*df* = 1)_ = 767.61, *p* < 0.001]; Doornik–Hansen omnibus test [χ^2^_(*df* = 18)_ = 266.69, *p* < 0.001]). Based on previous research (Bati et al., 2018; McElroy et al., 2019; Starcevic & Berle, 2013), we developed a model including health anxiety as a predictor of cyberchondria dimensions. Additionally, based on previous research (Barke et al., 2016; Bati et al., 2018; Fergus & Russell, 2016; Norr et al., 2015; Starcevic & Berle, 2013; Trinkhaus, 2019), we considered the mediation effects between health anxiety and cyberchondria via distress, pessimism, and difficulties in emotion regulation. The model also included correlations between the residuals of all cyberchondria dimensions. Moreover, we analyzed correlations between pessimism residual and distress residual and between difficulties in emotion regulation residual and distress residual. However, to ensure the clarity of the model, we excluded these correlations from Fig. 1; their values are provided in the supplementary materials.

The χ^2^, χ^2^/*df*, RMSEA (root mean square error of approximation), SRMR (standardized root mean square residual), CFI (comparative fit index), and TLI (Tucker–Lewis index) statistics were applied as measures of model fit (Hu & Bentler, 1999; Kline, 2011). Statistically non-significant χ^2^ values (*p* > 0.05) may suggest that the proposed model fits the dataset. If the χ^2^/*df* ratio is lower than 2, it suggests a good fit to the dataset. Likewise, values of RMSEA lower than 0.05 and values of SRMR lower than 0.08 show a good fit of the model. Values of CFI and TLI higher than 0.95 demonstrate that the model fits the dataset well (Hu & Bentler, 1999; Kline, 2011).

Moreover, to analyze the mediation effects between health anxiety (illness likelihood, negative consequences of illness) and cyberchondria (excessiveness, distress, reassurance, compulsion) via difficulties in emotion regulation, distress, and pessimism, we used the approach proposed by Zhao et al. (2010) involving the Monte Carlo method (5,000 samples) to estimate standardized indirect effects with 95% confidence intervals (Mehmetoglu, 2018). We interpreted the mediation effect in accordance with Zhao et al. (2010) guidelines: (1) complementary mediation: indirect effect and direct effect both exist and point in the same direction; (2) competitive mediation: indirect effect and direct effect both exist and point in opposite directions; (3) indirect-only mediation: indirect effect exists, but no direct effect (full mediation); (4) direct-only non-mediation: direct effect exists, but no indirect effect; and (5) no-effect non-mediation: neither direct nor indirect effect exists. The statistical calculations were conducted using IBM SPSS 23 statistical software for descriptive statistics and correlation analysis and Stata 14 with medsem.ado package (Mehmetoglu, 2018) for structural equation analysis and mediation analysis.

## Results

The results of descriptive analysis are presented in Table 1. Correlation analysis revealed positive correlations of all dimensions of cyberchondria with health anxiety: illness likelihood, health anxiety: negative consequences of illness, pessimism, distress, and difficulties in emotion regulation. Gender was significantly negatively related to cyberchondria total score and to two dimensions of cyberchondria: excessiveness and distress. There were also significant negative correlations between all dimensions of cyberchondria and age. Detailed results are presented in Table 1.

**Table 1:** Mean values, standard deviations, and correlations between the analyzed variables (*N* = 615)

Variables	*M*	*SD*	[1]	[2]	[3]	[4]	[5]	[6]	[7]	[8]	[9]	[10]	[11]
[1] Health anxiety: illness likelihood	25.00	6.76											
[2] Health anxiety: negative consequences of illness	8.87	2.36	.43***										
[3] Pessimism	15.47	4.00	.24***	.30***									
[4] Distress	14.10	4.85	.45***	.44***	.32***								
[5] DERS	79.20	20.44	.39***	.34***	.19***	.45***							
[6] Gender	0.47	0.50	− .04	− .12**	.04	− .17***	− .15***						
[7] Age	43.86	14.57	.04	.03	− .06	− .24***	− .18***	.03					
Cyberchondria													
[8] Excessiveness	8.92	2.47	.48***	.31***	.18***	.29***	.40***	− .16***	− .15***				
[9] Distress	7.04	2.73	.53***	.43***	.22***	.38***	.42***	− .14***	− .12**	.64***			
[10] Reassurance	7.11	2.53	.45***	.22***	.17***	.23***	.29***	− .05	− .09*	.63***	.63***		
[11] Compulsion	6.29	2.54	.42***	.39***	.30***	.34***	.36***	− .05	− .16***	.57***	.69***	.59***	
[12] Total	29.36	8.74	.56***	.40***	.25***	.37***	.44***	− .12**	− .16***	.83***	.88***	.83***	.83***

0 = female; 1 = male; *DERS* Difficulties in emotion regulation ***p < .001. **p < .01. *p < .05

Based on structural model analyses, we established that the analyzed model was a good fit to the data: χ^2^_(*df* = 1)_ = 0.82, *p* = 0.365; χ^2^/*df* = 0.82; RMSEA = 0.001, SRMR = 0.005, CFI = 1.000 and TLI = 1.004. The results revealed that health anxiety: illness likelihood was significantly positively related to all dimensions of cyberchondria (excessiveness: β = 0.35, *p* < 0.001; distress: β = 0.36, *p* < 0.001; reassurance: β = 0.39, *p* < 0.001; compulsion: β = 0.25, *p* < 0.001).

**Fig. 2:**
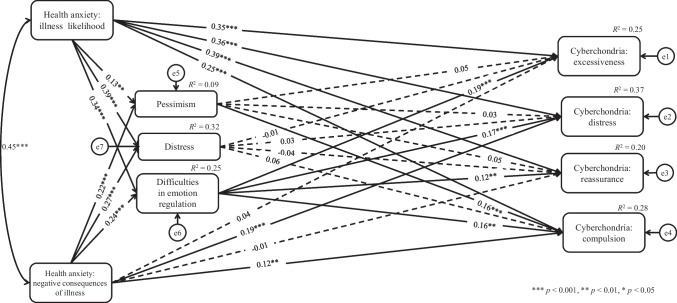
Path model of relations between the analyzed variables

Furthermore, pessimism (β = 0.13, *p* = 0.003), distress (β = 0.39, *p* < 0.001), and difficulties in emotion regulation (β = 0.34, *p* < 0.001) were positively related to health anxiety: illness likelihood. Health anxiety: negative consequences of illness was a variable significantly positively related to two dimensions of cyberchondria—namely, distress (β = 0.19, *p* < 0.001) and compulsion (β = 0.12, *p* = 0.010). It was also significantly positively related to pessimism (β = 0.22, *p* < 0.001), distress (β = 0.27, *p* < 0.001), and difficulties in emotion regulation (β = 0.24, *p* < 0.001). The results revealed a significant positive relationship between pessimism and cyberchondria: compulsion (β = 0.16, *p* < 0.001). Difficulties in emotion regulation were positively and significantly related to all dimensions of cyberchondria (excessiveness: β = 0.19, *p* < 0.001; distress: β = 0.17, *p* < 0.001; reassurance: β = 0.12, *p* = 0.005; compulsion: β = 0.16, *p* = 0.001). Detailed results are shown in Fig. 2.

Based on the mediation analysis framework (Mehmetoglu, 2018; Zhao et al., 2010), we found significant mediation effects of pessimism and difficulties in emotion regulation. More specifically, health anxiety: illness likelihood exerted a significant indirect effect on cyberchondria: compulsion via pessimism. Similarly, we found a standardized indirect effect of health anxiety: negative consequences of illness on cyberchondria: compulsion mediated by pessimism. Moreover, there were significant standardized indirect effects between health anxiety: illness likelihood and all cyberchondria dimensions via difficulties in emotion regulation. Given the statistically significant direct effects between health anxiety: illness likelihood and all cyberchondria dimensions, the results may indicate partial mediation. There were also significant standardized indirect effects between health anxiety: negative consequences of illness and all cyberchondria dimensions via difficulties in emotion regulation. Given the statistically significant direct effects between health anxiety: negative consequences of illness and cyberchondria: distress and between health anxiety: negative consequences of illness and cyberchondria: compulsion, these results may indicate partial mediation. However, given the statistically non-significant direct effects of this type of health anxiety on cyberchondria: excessiveness and cyberchondria: reassurance, these results may indicate full mediation. Detailed results are shown in Table 2.

**Table 2:** Standardized indirect effects with 95% confidence intervals

Model pathways	Point estimates	Standard error	95% CI		*z*	*p*
			Lower	Upper		
HAI ➔ pessimism ➔ CC	.020	.009	.005	.040	2.23	.026
HAI ➔ DER ➔ CE	.065	.019	.031	.105	3.47	.001
HAI ➔ DER ➔ CD	.058	.017	.026	.094	3.35	.001
HAI ➔ DER ➔ CR	.043	.016	.012	.076	2.59	.010
HAI ➔ DER ➔ CC	.053	.018	.019	.091	2.88	.004
HAN ➔ pessimism ➔ CC	.034	.013	.013	.062	2.72	.006
HAN ➔ DER ➔ CE	.046	.015	.020	.078	3.09	.002
HAN ➔ DER ➔ CD	.041	.014	.017	.070	3.00	.003
HAN ➔ DER ➔ CR	.030	.012	.008	.056	2.41	.016
HAN ➔ DER ➔ CC	.037	.014	.013	.068	2.65	.008

*HAI* Health anxiety: illness likelihood; *HAN* Health anxiety: negative consequences of illness; *DER* Difficulties in emotion regulation; *CE* Cyberchondria: excessiveness; *CD* Cyberchondria: distress; *CR* Cyberchondria: reassurance; *CC* Cyberchondria: compulsion

## Discussion

The main aim of the study was to determine the role of emotional functioning in the relationship between health anxiety and cyberchondria. We predicted that pessimism, distress, and difficulties in emotion regulation would be mediators between health anxiety and cyberchondria. Our results indicate a positive relationship between health anxiety and cyberchondria and the mediating role of pessimism and difficulties in emotion regulation in this relationship. We also found that participants high in cyberchondria tended to be younger and were more often female. The results of our study demonstrate that cyberchondria is negatively related to age. This means that younger women are particularly at risk and should be targeted by special prevention measures against the development of cyberchondria. The high incidence of cyberchondria among young people probably stems from the fact that they use the Internet more often than the older generation (Jiang & Song, 2022). Consequently, they are more likely to look for information about their health online (Lopez-Fernandez, 2019). It is therefore essential that the health information they find there is based on sound medical knowledge (McElroy & Shevlin, 2014). It is also important to teach Internet users to distinguish reliable content from false information, and this applies to all information that appears online, not just that related to health (Dumitru et al., 2022). We predicted that health anxiety would be related to cyberchondria. The study showed that higher health anxiety was associated with higher scores on all dimension of cyberchondria. This result is in line with previous research indicating associations between cyberchondria and health anxiety (McElroy et al., 2019; Starcevic & Berle, 2013). People who experience a higher level of anxiety are more at risk of illness and tend to attach greater importance to health-related information (Hadjistavropoulos et al., 1998; McMullan et al, 2019). Consequently, they probably more often search for such information on the Internet. Health anxiety makes a person alert to their health, and browsing the Internet is a quick and easy way to find information. This, however, can mean that the more one searches for information, the more possible illnesses one finds—which, paradoxically, raises the level of anxiety (Starcevic & Berle, 2013). This is also a clue for doctors—if they fail to provide patients with thorough answers to their health-related questions, the patients will be looking for more information about their health on the Internet, where they may come across both credible and non-credible sources (Alpaslan, 2016). Instead of providing reassurance, information obtained online will often increase health anxiety and lead to an increase in cyberchondria. This makes it essential that specialists (doctors, health care professionals, website administrators) cooperate; sites posting health information should be subject to verification, and those containing false or unreliable information should be either blocked or appropriately marked (e.g., Dumitru et al., 2022).

We predicted that pessimism would be a mediator in the relationship between health anxiety and cyberchondria. Our results indicate that pessimism is a mediator between health anxiety and cyberchondria: compulsion. They additionally indicate partial mediation of pessimism between health anxiety and cyberchondria. We also expected that the relationship between health anxiety and cyberchondria would be mediated by distress, but the results did not support this prediction. There are studies that can be cited as indicating that pessimism is associated with health anxiety (Bajcar & Babiak, 2020; Maftei & Holman, 2020; Reizer et al., 2022). Health anxiety translates into greater pessimism, which results in an increased tendency to search for health information on the Internet. Health anxiety induces a negative mindset, and any information about a possible illness leads to thoughts such as “I'm sure I'm going to be sick,” which in turn can trigger a negative spiral of emotions (health anxiety), thoughts (pessimism), and behavior (information seeking).

Moreover, we predicted that difficulties in emotion regulation would be a mediator in the relationship between health anxiety and cyberchondria. The study revealed that difficulties in emotion regulation partially mediated the relationships between health anxiety: negative consequences of illness and two dimensions of cyberchondria: distress and compulsion. They also fully mediated the relations between health anxiety: negative consequences of illness and two other dimensions of cyberchondria: excessiveness and reassurance. Previous research showed that problems with emotion regulation were strongly associated not only with anxiety but also with a fear of falling in the cognitive sense (i.e., concern about falling) and in the behavioral sense (i.e., avoidance of activity; Scarlett et al., 2019). Problems with emotion regulation result in maladaptive ways of coping with health anxiety, which in turn translate into seeking health information instead of medical consultation (Görgen et al., 2014; Singh & Brown, 2016). This means proper emotional regulation is a protective factor against excessive searching for health-related information on the Internet; it is also linked to low health anxiety. An indirect way to counteract cyberchondria may therefore be to improve emotional regulation skills in individuals—especially in young people, whom this study has found to be particularly likely to develop cyberchondria.

### Limitations and directions for future research

The research presented in this paper is not free from limitations. Firstly, the measures used were based on self-report, which makes it impossible to draw causal conclusions. Longitudinal and experimental studies should be conducted in the future. In our study we assumed that the subjects were healthy, but in further research on cyberchondria it would be advisable to collect information about participants’ health condition. Another interesting research direction would be to identify the motives for seeking health information. For example, it can be investigated whether people seek such information because they mistrust the health care system or whether they start to look for it upon seeing information about a particular disease on the Internet and concluding that their symptoms fit this pattern.

The results of our study show that cyberchondria is negatively related to age. This is probably due to the fact that young people use the Internet more often than older generations. Future research should therefore include variables associated with Internet skills and with access to the Internet. Another point to note is that the present study was conducted before the COVID-19 pandemic. It would be useful to consider similar studies conducted during the increase in morbidity and mortality and after the pandemic (Zheng et al., 2020), especially as recent results suggest that cyberchondria and health anxiety are strongly related to coronavirus anxiety (Jungmann & Witthöft, 2020).

The presented research results may inspire further explorations into cyberchondria. An interesting direction for future study would be to identify the motives for seeking health information on the Internet. Researchers may want to determine if such behavior is caused by poor medical care, a fear of visiting the doctor, the experience of illness in the family, or information found online.

## Conclusions

The topic of cyberchondria seems to be an important one to investigate because Internet forums provide not only verified materials, reliably reflecting the current state of knowledge, but also inaccurate or misleading entries and comments posted by non-specialists. There are, naturally, plenty of reliable and at the same time accessible texts on the Internet, but these often prove to be difficult to distinguish from non-credible sources.

In some individuals, constant thinking about health can cause not only concern for their own and their family's health and living conditions but also automatic thoughts related to anxiety and worry. This phenomenon has been termed habitual anxious worrying (Papageorgiou, 2006). Paradoxically, anxious worrying can also affect patients who work intensively with medical staff and who have significantly greater knowledge about their condition than average individuals. The presented results show that emotional functioning variables—namely, pessimism and difficulties in emotion regulation—play an important role in the relationship between health anxiety and cyberchondria.

## Supplementary Information

Below is the link to the electronic supplementary material.

Supplementary file1 (DOCX 14.9 KB)

## Data Availability

The datasets generated during and/or analyzed during the current study are available from the corresponding author on reasonable request.
