# Supplementary material for: The role of emotional functioning in the relationship between health anxiety and cyberchondria
Source: Curr Psychol. 2022 Dec 19:1–11. Online ahead of print. doi: 10.1007/s12144-022-04126-3 (PMC9762653; doi:10.1007/s12144-022-04126-3)
Supplement: Supplementary file 1 — Supplementary file1 (DOCX 14.9 KB) [file 12144_2022_4126_MOESM1_ESM.docx]

Table A. Correlation between residuals of variables.

| Variable 1 | Variable 2 | r | SE | z | p | 95%CI | 95%CI |
| --- | --- | --- | --- | --- | --- | --- | --- |
| Cyberchondria-Excessiveness | Cyberchondria-Distress | 0.51 | 0.03 | 14.73 | 0.001 | 0.44 | 0.57 |
| Cyberchondria-Excessiveness | Cyberchondria-Reassurance | 0.55 | 0.03 | 17.51 | 0.001 | 0.49 | 0.61 |
| Cyberchondria-Excessiveness | Cyberchondria-Compulsion | 0.43 | 0.04 | 10.39 | 0.001 | 0.35 | 0.51 |
| Cyberchondria-Distress | Cyberchondria-Reassurance | 0.52 | 0.03 | 15.22 | 0.001 | 0.46 | 0.59 |
| Cyberchondria-Distress | Cyberchondria-Compulsion | 0.59 | 0.03 | 16.89 | 0.001 | 0.52 | 0.66 |
| Cyberchondria-Reassurance | Cyberchondria-Compulsion | 0.50 | 0.04 | 13.94 | 0.001 | 0.43 | 0.58 |
| Optimism | Distress | 0.15 | 0.04 | 3.69 | 0.001 | 0.07 | 0.23 |
| Distress | Difficulties in emotion regulation | 0.29 | 0.04 | 7.29 | 0.001 | 0.21 | 0.36 |
